# Household air pollution and arthritis in low-and middle-income countries: Cross-sectional evidence from the World Health Organization’s study on Global Ageing and Adult Health

**DOI:** 10.1371/journal.pone.0226738

**Published:** 2019-12-27

**Authors:** Shelby S. Yamamoto, Elaine Yacyshyn, Gian S. Jhangri, Arvind Chopra, Divya Parmar, C. Allyson Jones

**Affiliations:** 1 School of Public Health, University of Alberta, Edmonton, Canada; 2 Division of Rheumatology, Faculty of Medicine & Dentistry, University of Alberta, Edmonton, Canada; 3 Centre for Rheumatic Diseases, Pune, India; 4 School of Health Sciences, City, University of London, London, England, United Kingdom; 5 Faculty of Rehabilitation Medicine, University of Alberta, Edmonton, Canada; University of British Columbia, CANADA

## Abstract

**Background:**

Evidence points to a clear link between air pollution exposure and several chronic diseases though investigations regarding arthritis are still lacking. Emerging evidence suggests an association between ambient air pollution and rheumatoid arthritis. Household air pollution exposure, conversely, is largely unstudied but may be an important consideration for arthritis, particularly in low- and middle-income countries (LMICs), where cooking and heating activities can generate high indoor air pollutant levels.

**Methods:**

We investigated the association of household air pollution (electricity vs. gas; kerosene/paraffin; coal/charcoal; wood; or agriculture/crop/animal dung/shrubs/grass as the main fuel used for cooking) and arthritis in six LMICs (China, Ghana, India, Mexico, the Russian Federation, South Africa) using data from Wave I of the World Health Organization Study on Global AGEing and Adult Health (SAGE) (2007–2010). Multivariable analyses were adjusted for sociodemographic, household and lifestyle characteristics and several comorbidities.

**Results:**

The use of gas (aOR = 1.76, 95%CI: 1.40–2.21); coal (aOR = 1.74, 95%CI: 1.22–2.47); wood (aOR = 1.69, 95%CI: 1.30–2.19); or agriculture/crop/animal dung/shrubs/grass: aOR = 1.95 (1.46–2.61) fuels for cooking were strongly associated with an increased odds of arthritis, compared to electricity in cluster and stratified adjusted analyses. Gender (female), age (≥50 years), overweight (25.0 ≤BMI<30.0 kg/m^2^), obesity (BMI ≥30.0 kg/m^2^), former and current alcohol consumption, and the comorbidities angina pectoris, diabetes, chronic lung disease, depression and hypertension were also associated with a higher odds of arthritis. Underweight (BMI<18.5 kg/m^2^) and higher education levels (college/university completed/post-graduate studies) were associated with a lower odds of arthritis.

**Conclusions:**

These findings suggest that exposure to household air pollution from cook fuels is associated with an increased odds of arthritis in these regions, which warrants further investigation.

## Background

Household air pollution continues to be a public health issue, particularly for those in low- and middle-income countries (LMICs).[1] A significant source of household air pollution is generated from cooking and heating activities that rely on solid biomass fuels.[2–5] Approximately 3 billion people globally still largely rely on solid biomass fuels.[6]

Air pollution is associated with number of chronic adverse health effects, including non-communicable diseases such as cardiovascular disease, chronic obstructive pulmonary disease and lung cancer.[1, 7–12] However, very few studies have explored the impact of air pollution on arthritis, particularly in LMIC settings.[13–18] Though the age standardized prevalence of osteoarthritis (OA) and rheumatoid arthritis (RA) in areas such as South and East Asia have been estimated to be lower than in North America, the disability-adjusted life years (DALYs), a measure of disease burden, is often much higher in these regions.[19, 20] For example, the mean DALYs for hip and knee OA (2010) in South Asia were estimated to be 2,466 (per 100,000) compared to 1,117 (per 100,000) in North America.[19] The burden of OA, specifically, is exacerbated in LMICs as a result of ageing populations, lack of access to preventative and therapeutic interventions and greater numbers of people with moderate to severe forms of the disease.[19, 21, 22] Similarly, DALYs for RA (2010) for women in South Asia were estimated to be 445 (per 1,000) and 338 (per 1,000) for women in North America.[20] Global estimates may also be underestimated due to the underdiagnosis of the condition in some LMIC settings.[20, 23, 24]

Most OA and RA studies have focused on smoking as an important environmental risk factor.[25–28] However, emerging evidence from high-income countries (HICs) suggests that ambient air pollution exposure is also a risk factor for RA.[13–18] Investigations concerning household air pollution and OA have not yet been undertaken. Household air pollution may similarly be an important risk factor in LMICs where indoor air pollution levels can be high,[5] though this is currently unexplored. Proposed biological pathways for the air pollution-RA relationship could be related to oxidative stress and immune suppression, similar to those mechanisms proposed for smoking.[16, 29, 30] Pathways linking OA and air pollution are currently unexplored. Cartilage loss from smoking may be associated with OA progression.[31, 32] The aim of this study was to examine the association between household air pollution and arthritis in LMICs.

## Methods

### Sample

Data from wave I of the multiwave panel World Health Organization (WHO) Study on Global AGEing and Adult Health (SAGE) (2007–2010) was used for this study.[33] Data from face-to-face interviews were obtained from standardized questionnaires in six LMICs: China (n = 13,656), Ghana (n = 4,776), India (n = 10,829), Mexico (n = 2,349), the Russian Federation (n = 3,640) and South Africa (n = 3,064). Questionnaires were translated according to the World Health Survey protocol and the validity and reliability of the instruments were tested in a pilot survey across data collection sites.[34] Nationally representative samples were selected from populations in these countries and included those aged ≥18 years, with a particular focus on those ≥50 years old.[33] A stratified multistage cluster sampling design was used with household-level analysis weights and person-level analysis weights calculated and post-stratified for each country.[33] Further information regarding the study, sampling approach and weighting scheme used in SAGE is published elsewhere.[33, 35]

### Outcome

The outcome in this study, arthritis (yes, no), was derived using the algorithm of Arokiasamy et al, based on several SAGE survey questions.[36] Participants were considered to have arthritis if they responded yes to the questions: “Have you ever been diagnosed with/told you have arthritis (a disease of the joints, or by other names rheumatism or osteoarthritis)?” or “Have you been taking medications or other treatment for it during the last 12 months?”[36, 37] Participants were also coded as having arthritis if they responded yes to questions 1, 2 and 4 and “About 30 minutes or less” to question 3: (Q1) “During the last 12 months, have you experienced, pain, aching, stiffness or swelling in or around the joints (like arms, hands, legs or feet) which were not related to an injury and lasted for more than a month?”; (Q2) “During the last 12 months, have you experienced stiffness in the joint in the morning after getting up from bed, or after a long rest of the joint without movement?”; (Q3) “How long did this stiffness last?”; and (Q4) “Did this stiffness go away after exercise or movement in the joint?”[36] As the determination of the outcome relied on self-reports and symptoms, we were unable to distinguish between different types of arthritis.

### Exposure

Exposure to household air pollution was assessed in a question that asked participants about the main type of fuel used in the household for cooking. A series of eight options were provided, which were further grouped into (1) electricity; (2) gas; (3) kerosene/paraffin; (4) coal/charcoal; (5) wood; and (6) agriculture/crop/animal dung/shrubs/grass for this study as strata counts for some options were small.

### Covariates

A number of covariates identified *a priori* from the literature were also examined in this study. Socio-demographic characteristics included: age (18–49, 50–59, 60–69 and ≥70 years); gender (male, female); marital status (currently married/cohabitating, never married/separated/divorced/widowed); highest education level (primary school or less, secondary school completed, high school or equivalent completed, and college/university completed/post-graduate studies), body mass index (BMI) (underweight: BMI<18.5 kg/m^2^, normal weight: 18.5≤BMI<25.0 kg/m^2^, overweight: 25.0≤BMI<30.0 kg/m^2^, obese: BMI≥30.0 kg/m^2^), setting (urban, rural), and country (China, Ghana, India, Mexico, Russia, South Africa). Household income was assessed using a combination of assets, income, household expenditures (food, goods, services, health care), which were included in this study as quintiles (1: lowest to 5: highest).[33]

Lifestyle characteristics included tobacco use, alcohol use and physical activity. Tobacco use was assessed as never, former or current user. Similarly, alcohol use was also classified as never, former and current drinker. Leisure, occupation-related and transport-related physical activity was assessed in the survey and grouped into: <75 minutes of vigorous or <150 minutes of moderate activity per week; or ≥75 minutes of vigorous or ≥150 minutes of moderate activity per week, according to the WHO Global Recommendations on Physical Activity for Health.[38]

Several comorbidities (angina, diabetes, chronic lung disease (emphysema, bronchitis, chronic obstructive pulmonary disease), asthma, depression, hypertension and stroke) were assessed using questions from the survey. The algorithms of Arokiasamy et al.[36] were similarly applied to code participants as not afflicted (no) or afflicted (yes).

### Statistical analyses

Data were first examined descriptively with percentages presented for categorical variables, median and IQR (interquartile range) for continuous variables, by country. Pooled data were also examined. Complete case analysis using multivariable logistic regression was conducted to investigate the relationship between household air pollution and arthritis status. Models were constructed by sequentially adding the blocks of covariates previously identified and described starting with a base model of individual and household risk factors (socio-demographic plus household). Model 2 incorporated lifestyle characteristics and model 3 included comorbidities. Sensitivity analyses were run by country (solid vs non-solid fuels). Further examinations also examined the two countries with the highest proportion of electricity users for cooking (Russia and South Africa) and consistent results were obtained (data not shown). Analyses stratified by gender were also conducted to investigate potential effect modification. Interactions were similarly examined in pooled analyses. Strata and cluster variables (primary sampling units) provided in the dataset were incorporated into the analyses to account for the stratified, multistage clustered sampling design of the study. Post-stratified person-level weights, based on sample selection and a post-stratification factor, were included to account for the study sampling design in the analyses.[33] All analyses were performed using SAS 9.4.

### Ethical approval and informed consent

The SAGE study received ethical approval from the World Health Organization Ethical Review Committee and organizations in each country in the study prior to data collection. Informed written consent was sought and obtained from all participants.[34]

## Results

### Pooled and country-level participant characteristics

Education levels varied widely between the countries, with Russia reporting the highest levels of education and India the lowest (Table 1). The prevalence of overweight participants was highest in Mexico (49.4%) with obesity prevalence the highest in South Africa (29.6%). Current tobacco use was most frequently reported in India, followed by China. Russia had the highest proportion of people with current use of alcohol (53%).

**Table 1:** Characteristics of participants by country and pooled*.

Characteristics %		China (N = 13,656)	Ghana (N = 4,776)	India (N = 10,829)	Mexico (N = 2,349)	Russia (N = 3,640)	South Africa (N = 3,064)	Total pooled (N = 38,314)
**Socio-demographic**								
Gender								
	Male	50.9	50.1	51.2	47.8	43.6	44.7	49.9
	Female	49.1	49.9	48.8	52.2	56.4	55.3	50.1
Age (years)								
	18 to 49	74.6	75.4	75.3	73.2	58.5	75.0	73.0
	50 to 59	11.5	9.9	12.2	13.6	19.4	12.5	12.7
	60 to 69	8.2	6.8	7.6	6.9	10.1	7.6	8.1
	≥70	5.8	7.9	4.9	6.4	11.9	4.9	6.2
	Median (years)	44	42	40	39	46	43	43
	Interquartile range (IQR)	38–50	35–49	30–49	32–51	36–58	32–50	35–51
Marital status								
	Currently married/cohabitating	89.4	73.1	82.1	70.6	60.5	52.0	81.5
	Never/separated/divorced/widowed	10.6	26.9	17.9	29.4	39.5	48.0	18.5
Education								
	Primary school or less	38.2	63.6	61.2	52.4	3.1	36.2	43.9
	Secondary school completed	32.5	10.8	15.8	23.4	10.1	27.0	22.3
	High school (or equivalent) completed	20.6	21.0	14.8	11.8	64.3	29.4	23.7
	College/university completed/post-graduate	8.7	4.5	8.2	12.4	22.6	7.5	10.1
BMI classification (kg/m^2^)								
	Underweight (BMI<18.5)	4.2	9.1	36.0	0.9	1.5	3.0	17.2
	Normal (18.5≤BMI<25.0)	63.6	57.1	52.4	21.2	41.2	36.8	55.3
	Overweight (25.0≤BMI<30.0)	27.5	21.1	9.0	49.4	36.8	30.5	20.9
	Obese (BMI≥30.0)	4.8	12.6	2.6	28.6	20.5	29.6	6.7
**Household**								
Primary cook fuel type								
	Electricity	4.9	0.1	0.1	0.9	18.6	75.8	6.7
	Gas	50.5	8.9	19.3	90.8	79.7	4.3	38.9
	Kerosene/paraffin	0.0	0.4	0.9	0.0	0.1	11.2	0.8
	Coal/charcoal	4.1	33.9	2.4	0.1	0.1	0.3	3.3
	Wood	12.6	54.9	60.3	8.2	1.5	8.1	31.7
	Agriculture/crop/animal dung/shrub/grass	27.8	1.7	17.0	0.0	0.0	0.2	18.7
Income quintile								
	1^st^ (lowest—poorest)	9.9	15.3	20.7	15.4	12.2	17.2	15.0
	2^nd^	16.3	17.0	21.1	24.4	12.7	20.0	18.0
	3^rd^	18.9	19.8	20.0	20.8	13.5	22.6	18.8
	4^th^	23.8	22.3	18.1	13.8	25.3	20.8	21.5
	5^th^ (highest—richest)	31.1	25.6	20.1	25.6	36.3	19.4	26.7
Residential area								
	Urban	47.7	45.3	25.3	76.3	80.5	73.1	43.1
	Rural	52.3	54.7	74.7	23.7	19.5	26.9	56.9
**Lifestyle**								
Tobacco use								
	Never	63.6	84.1	54.9	58.2	60.6	65.6	60.0
	Former	3.5	7.7	2.3	16.1	13.4	7.4	4.4
	Current	32.9	8.2	42.8	25.7	26.0	27.1	35.6
Alcohol consumption								
	Never	64.1	44.8	82.9	41.8	17.0	72.8	66.2
	Former	9.1	24.0	8.3	26.8	29.2	9.0	11.4
	Current	26.8	31.2	8.9	31.5	53.8	18.2	22.4
Leisure time physical activity								
	<75 minutes vigorous or <150 minutes moderate activity per week	84.5	85.4	83.6	89.5	76.9	88.1	83.3
	≥75 minutes vigorous or ≥150 minutes moderate activity per week	15.5	14.6	16.4	10.5	23.1	11.9	16.7
Occupation-related physical activity								
	<75 minutes vigorous or <150 minutes moderate activity per week	41.8	23.3	25.2	59.8	19.0	49.7	32.1
	≥75 minutes vigorous or ≥150 minutes moderate activity per week	58.2	76.7	74.8	40.2	81.0	50.3	67.9
Transport-related physical activity								
	<75 minutes vigorous or <150 minutes moderate activity per week	58.3	41.1	40.0	55.0	31.2	72.9	47.6
	≥75 minutes vigorous or ≥150 minutes moderate activity per week	41.7	58.9	60.0	45.0	68.8	27.1	52.4
**Co-morbidities**								
	Arthritis	15.9	14.0	15.2	7.9	23.2	12.4	16.4
	Angina pectoris	4.0	2.7	5.5	2.1	17.4	3.1	6.2
	Diabetes	2.8	2.2	3.2	10.3	3.6	3.4	3.1
	Chronic lung disease	5.7	2.3	10.1	14.7	15.7	5.1	8.7
	Asthma	2.0	2.8	6.8	2.3	3.4	3.9	4.3
	Depression	1.4	5.1	11.7	11.9	5.2	7.3	6.4
	Hypertension	37.8	43.9	24.3	12.2	42.7	54.5	33.4
	Stroke	0.9	1.3	1.0	1.8	2.4	1.6	1.1

* Post-stratified weighted, by country

### Pooled multivariable analyses

#### Participant characteristics by gender

The median age of men and women in the study was 42.7 (IQR: 34.4–49.8) and 42.1 (IQR: 33.4–51.4) years, respectively. Obesity (BMI≥30.0 kg/m^2^) was higher among women compared to men (9.2% and 4.2%, respectively), though the proportion of overweight was similar (20.9% for both). Sixty percent of men compared to 11.3% of women reported current use of tobacco products. Current alcohol consumption was 35.8% among men and 9.1% among women (descriptive participant characteristics by gender not shown).

#### Arthritis

Overall, arthritis (16.4%) was the second most prevalent condition, after hypertension (33.4%) (Table 1). Of all the countries, the prevalence of arthritis was highest in Russia (23.2%) followed by China (15.9%) and India (15.2%) (Table 1). The prevalence of arthritis was higher in women (19.5%) compared to men (13.2%). Among the four age groups, the prevalence of arthritis was highest among those ≥70 years (Table 2). In those with less education, a higher prevalence of arthritis was also observed. The distribution of arthritis was similar between income quintiles. A higher prevalence of arthritis was observed among those that were overweight and obese. The prevalence of arthritis was also higher among those with comorbid conditions (Table 2).

**Table 2:** Multivariable model examining variables associated with arthritis in pooled sample (N = 38,314)*.

			Arthritis	Model 1**			Model 2***			Model 3****		
Characteristics			%	OR	95% CI	p-value	OR	95% CI	p-value	OR	95% CI	p-value
**Socio-demographic**												
Gender												
	Male		13.2	1.00			1.00			1.00		
	Female		19.5	1.46	1.27–1.69	<0.001	1.72	1.45–2.03	<0.001	1.77	1.49–2.09	<0.001
Age (years)												
	18 to 49		11.5	1.00			1.00			1.00		
	50 to 59		25.5	2.30	2.01–2.64	<0.001	2.33	2.03–2.67	<0.001	2.06	1.79–2.37	<0.001
	60 to 69		31.1	3.06	2.67–3.50	<0.001	3.11	2.73–3.54	<0.001	2.48	2.15–2.86	<0.001
	≥70		35.5	3.70	3.18–4.31	<0.001	3.84	3.31–4.46	<0.001	2.78	2.36–3.28	<0.001
Marital status												
		Currently married/cohabitating	16.0	1.00			1.00			1.00		
		Never/separated/divorced/widowed	18.0	0.91	0.76–1.09	0.31	0.91	0.76–1.09	0.31	0.91	0.74–1.09	0.29
Education												
		Primary school or less	19.4	1.00			1.00			1.00		
		Secondary school completed	15.4	0.93	0.75–1.15	0.49	0.96	0.77–1.18	0.69	0.93	0.76–1.15	0.52
		High school (or equivalent) completed	15.3	0.78	0.62–0.98	0.03	0.81	0.65–1.02	0.07	0.82	0.66–1.01	0.07
		College/university completed/post-graduate	7.9	0.41	0.29–0.59	<0.001	0.44	0.31–0.62	<0.001	0.45	0.32–0.64	<0.001
BMI classification (kg/m^2^)												
		Underweight (BMI<18.5)	13.5	0.77	0.66–0.91	0.002	0.77	0.66–0.91	0.002	0.75	0.64–0.89	<0.001
		Normal (18.5≤BMI<25.0)	14.6	1.00			1.00			1.00		
		Overweight (25.0≤BMI<30.0)	19.1	1.28	1.10–1.50	0.001	1.29	1.11–1.50	0.001	1.20	1.03–1.40	0.02
		Obese (BMI≥30.0)	30.4	2.02	1.57–2.59	<0.001	2.05	1.61–2.61	<0.001	1.70	1.31–2.21	<0.001
**Household**												
Primary cook fuel type												
		Electricity	12.2	1.00			1.00			1.00		
		Gas	16.7	1.58	1.22–2.04	<0.001	1.65	1.27–2.14	<0.001	1.74	1.33–2.27	<0.001
		Kerosene/paraffin	10.2	0.94	0.51–1.72	0.83	0.94	0.51–1.73	0.85	1.08	0.59–1.98	0.80
		Coal/charcoal	15.3	1.66	1.12–2.48	0.01	1.72	1.17–2.54	0.006	1.81	1.23–2.67	0.003
		Wood	15.8	1.66	1.23–2.24	0.001	1.69	1.25–2.28	<0.001	1.72	1.26–2.33	<0.001
		Agriculture/crop/animal dung/shrub/grass	18.5	1.90	1.39–2.62	<0.001	1.93	1.41–2.65	<0.001	1.96	1.41–2.71	<0.001
Income quintile												
		1^st^ (lowest—poorest)	18.3	1.00			1.00			1.00		
		2^nd^	16.4	0.87	0.69–1.10	0.24	0.88	0.70–1.10	0.26	0.89	0.71–1.13	0.35
		3^rd^	17.3	1.00	0.83–1.20	0.98	1.00	0.83–1.20	0.97	1.00	0.82–1.21	0.96
		4^th^	16.9	0.97	0.81–1.16	0.74	0.97	0.81–1.17	0.76	1.01	0.83–1.21	0.96
		5^th^ (highest—richest)	14.2	0.90	0.73–1.13	0.37	0.90	0.72–1.12	0.34	0.94	0.75–1.19	0.61
Setting												
		Urban	15.8	1.00						1.00		
		Rural	16.8	1.05	0.88–1.25	0.59	1.02	0.85–1.23	0.84	1.05	0.86–1.26	0.65
Country												
		China	15.9	1.00			1.00			1.00		
		Ghana	14.0	0.81	0.62–1.05	0.11	0.78	0.59–1.03	0.08	0.78	0.60–1.02	0.07
		India	15.2	1.07	0.88–1.30	0.53	1.16	0.95–1.43	0.15	0.98	0.80–1.20	0.85
		Mexico	7.9	0.36	0.25–0.51	<0.001	0.33	0.23–0.47	<0.001	0.30	0.20–0.44	<0.001
		Russia	23.2	1.61	1.22–2.12	<0.001	1.32	0.98–1.78	0.07	1.05	0.75–1.48	0.77
		South Africa	12.4	0.94	0.67–1.31	0.70	0.96	0.68–1.35	0.81	0.96	0.67–1.37	0.81
**Lifestyle**												
Tobacco use												
		Never	17.0				1.00			1.00		
		Former	18.0				1.06	0.78–1.43	0.72	0.96	0.71–1.31	0.81
		Current	15.2				1.07	0.93–1.24	0.32	1.05	0.91–1.21	0.50
Alcohol consumption												
		Never	15.6				1.00			1.00		
		Former	20.1				1.35	1.07–1.69	0.01	1.28	1.02–1.60	0.04
		Current	16.6				1.47	1.21–1.80	<0.001	1.50	1.22–1.84	<0.001
Leisure time physical activity												
		<75 minutes vigorous or <150 minutes moderate activity per week	17.3				1.00			1.00		
		≥75 minutes vigorous or ≥150 minutes moderate activity per week	11.7				0.87	0.68–1.12	0.28	0.89	0.69–1.13	0.33
Occupation-related physical activity												
		<75 minutes vigorous or <150 minutes moderate activity per week	16.2				1.00			1.00		
		≥75 minutes vigorous or ≥150 minutes moderate activity per week	16.5				1.05	0.90–1.22	0.54	1.09	0.93–1.28	0.28
Transport-related physical activity												
		<75 minutes vigorous or <150 minutes moderate activity per week	17.7				1.00			1.00		
		≥75 minutes vigorous or ≥150 minutes moderate activity per week	15.1				0.92	0.81–1.04	0.17	0.96	0.85–1.08	0.49
**Co-morbidities**												
Angina pectoris												
		No	14.6							1.00		
		Yes	43.2							2.37	1.76–3.18	<0.001
Diabetes												
		No	15.9							1.00		
		Yes	32.2							1.37	1.06–1.78	0.02
Chronic lung disease												
		No	14.7							1.00		
		Yes	34.4							1.76	1.43–2.16	<0.001
Asthma												
		No	15.6							1.00		
		Yes	33.1							1.28	0.997–1.64	0.05
Depression												
		No	15.2							1.00		
		Yes	34.3							2.31	1.90–2.80	<0.001
Hypertension												
		No	12.9							1.00		
		Yes	23.3							1.24	1.08–1.42	0.002
Stroke												
		No	16.2							1.00		
		Yes	31.2							1.10	0.82–1.46	0.53

* Post-stratified person-level weights applied

** Model 1 includes socio-demographic and household characteristics

*** Model 2 includes Model 1 characteristics and lifestyle characteristics

**** Model 3 includes Model 2 characteristics and comorbidities

#### Household air pollution

Overall, gas and wood were the primary fuels used most often by the households for cooking (38.9% and 31.7%, respectively). Mexico had the highest proportion of gas users (90.8%). South Africa reported the highest proportion of participants using electricity for cooking (Table 1). The proportion using wood for cooking was greatest in India, while China reported the highest proportion using agriculture/crop/animal dung/shrub/grass for cooking. The prevalence of arthritis was lowest among those using kerosene/paraffin for cooking (Table 2). Conversely, the prevalence of arthritis was highest among those using agriculture/crop/animal dung/shrub/grass products as cooking fuel.

In adjusted analyses, the type of primary cooking fuel used by the household was associated with arthritis in this study. Compared to electricity, all other forms of fuel, except kerosene/paraffin were associated with an increased odds of arthritis. The highest odds of arthritis were observed among those who used agriculture/crop/animal dung/shrub or grass fuels (Table 2).

Age and gender were also strongly associated with arthritis in the study. Women were much more likely to have arthritis compared to men (Table 2). The odds of arthritis also increased across age groups, with those in the highest age category (≥70 years) 2.78 times as likely to have arthritis compared to those in the youngest age category (18–49) (trend test: p = 0.002). Only the highest level of education (college/university completed/post-graduate studies) was associated with a lower odds of arthritis compared to primary or less education (aOR = 0.45, 95%CI: 0.32–0.64, trend test: p<0.001). Underweight (BMI<18.5 kg/m^2^) was associated with a decreased odds of arthritis (aOR = 0.75, 95%CI: 0.64–0.89) whereas obesity was associated with an increased odds of arthritis (aOR = 1.70, 95%CI: 1.31–2.21) compared to those of normal weight. Urbanicity was not found to be associated with arthritis. Participants in Mexico were much less likely to report having arthritis. When in explored in further analyses (solid vs non-solid fuels) by country, the association was no longer apparent. Former and current alcohol use was also associated with an increased odds of arthritis, compared to never drinkers. Participants with arthritis were also more likely to have comorbidities including angina pectoris, diabetes, chronic lung disease, depression and hypertension.

#### Gender-stratified multivariable analyses

Compared to electricity, the use of gas, wood and agriculture/crop/animal dung/shrub/grass as primary cooking fuels were associated with an increased odds of arthritis in men. Among women, the use of gas, coal/charcoal and agriculture/crop/animal dung/shrub/grass as primary cooking fuels were linked to a higher odds of arthritis compared to electricity (Table 3). Higher odds of arthritis with the use of most fuel types were observed among men as compared to women. In both men and women, older ages were also strongly associated with a higher odds of arthritis (Table 3). College or university level education or higher was associated with a lower odds of arthritis among both men and women, compared to primary or less. A negative association with arthritis among underweight and a positive association with arthritis among overweight and obese participants was only observed in women. Current tobacco use and alcohol consumption was associated with an increased odds of arthritis in women compared to those who never smoked or drank. A positive association between former and current alcohol use and arthritis was observed in men compared to never users. Comorbidities angina pectoris, chronic lung disease, depression and hypertension were associated with an increased odds of arthritis among both men and women. Among women, diabetes was also associated with a higher odds of arthritis.

**Table 3:** Multivariable model examining variables associated with arthritis by gender (N = 38,314)*.

			Men (N = 16,397)				Women (N = 21,917)			
Variable		Arthritis %	OR	95% CI	p-value	Arthritis %	OR	95% CI	p-value
**Socio-demographic**									
Age (years)									
	18 to 49	4.7	1.00			6.8	1.00		
	50 to 59	10.5	2.11	1.66–2.67	<0.001	15.0	2.03	1.67–2.47	<0.001
	60 to 69	12.1	2.42	1.95–3.00	<0.001	19.0	2.44	1.99–2.99	<0.001
	≥70	13.3	3.01	2.34–3.88	<0.001	22.2	2.54	2.04–3.18	<0.001
Marital status									
	Currently married/cohabitating	7.3	1.00			8.7	1.00		
	Never/separated/divorced/widowed	3.5	0.69	0.48–1.01	0.05	14.4	1.04	0.79–1.38	0.78
Education									
	Primary school or less	6.9	1.00			12.5	1.00		
	Secondary school completed	7.9	1.06	0.78–1.44	0.69	7.5	0.81	0.67–0.99	0.03
	High school (or equivalent) completed	5.7	0.75	0.59–0.97	0.03	9.6	0.85	0.64–1.12	0.25
	College/university completed/post-graduate	4.2	0.52	0.33–0.82	0.004	3.8	0.39	0.23–0.64	<0.001
BMI classification (kg/m^2^)									
	Underweight (BMI<18.5)	6.3	0.80	0.58–1.09	0.16	7.2	0.72	0.60–0.86	<0.001
	Normal (18.5≤BMI<25.0)	6.4	1.00			8.1	1.00		
	Overweight (25.0≤BMI<30.0)	7.2	1.10	0.84–1.43	0.50	11.9	1.27	1.03–1.56	0.03
	Obese (BMI≥30.0)	6.6	1.43	0.84–2.46	0.19	23.7	1.86	1.42–2.43	<0.001
**Household**									
Cook fuel type									
	Electricity	3.2	1.00			9.0	1.00		
	Gas	5.8	2.11	1.37–3.24	<0.001	11.0	1.54	1.15–2.06	0.004
	Kerosene/paraffin	2.7	0.87	0.31–2.40	0.79	7.4	1.26	0.65–2.46	0.49
	Coal/charcoal	3.9	1.37	0.85–2.20	0.19	11.3	2.05	1.09–3.87	0.03
	Wood	7.6	2.50	1.60–3.91	<0.001	8.2	1.31	0.91–1.87	0.15
	Agriculture/crop/animal dung/shrub/grass	8.4	2.48	1.57–3.93	<0.001	10.1	1.65	1.18–2.32	0.004
Income quintile									
	1^st^ (lowest—poorest)	7.9	1.00			10.3	1.00		
	2^nd^	6.6	0.80	0.58–1.11	0.18	9.8	0.97	0.74–1.26	0.80
	3^rd^	7.4	0.90	0.66–1.23	0.51	10.0	1.03	0.84–1.27	0.76
	4^th^	6.4	0.83	0.61–1.12	0.22	10.5	1.11	0.90–1.38	0.33
	5^th^ (highest—richest)	5.5	0.89	0.66–1.19	0.43	8.8	1.00	0.76–1.30	0.97
Setting									
	Urban	5.0	1.00			10.8	1.00		
	Rural	7.8	1.17	0.81–1.70	0.40	9.1	0.99	0.78–1.24	0.90
Country									
	China	9.5	1.00			15.6	1.00		
	Ghana	10.4	0.76	0.52–1.09	0.14	13.7	0.73	0.48–1.12	0.15
	India	7.9	0.83	0.58–1.19	0.32	13.4	1.02	0.80–1.31	0.87
	Mexico	4.6	0.23	0.13–0.38	<0.001	12.6	0.34	0.20–0.57	<0.001
	Russia	10.6	1.17	0.71–1.93	0.54	29.2	0.98	0.67–1.43	0.90
	South Africa	7.2	0.95	0.52–1.74	0.87	20.0	0.85	0.57–1.27	0.43
**Lifestyle**									
Tobacco use									
	Never	3.3	1.00			13.7	1.00		
	Former	13.2	0.83	0.59–1.17	0.30	4.7	1.12	0.66–1.89	0.68
	Current	11.4	0.91	0.77–1.09	0.30	3.8	1.25	1.01–1.55	0.04
Alcohol consumption									
	Never	4.3	1.00			11.4	1.00		
	Former	10.7	1.35	1.05–1.73	0.02	9.4	1.14	0.77–1.70	0.51
	Current	11.2	1.40	1.16–1.68	<0.001	5.4	1.67	1.12–2.50	0.01
Leisure time physical activity									
	<75 minutes vigorous or <150 minutes moderate activity per week	6.8	1.00			10.5	1.00		
	≥75 minutes vigorous or ≥150 minutes moderate activity per week	5.3	0.84	0.61–1.16	0.28	6.3	0.97	0.65–1.46	0.90
Occupation-related physical activity									
	<75 minutes vigorous or <150 minutes moderate activity per week	6.8	1.00			9.3	1.00		
	≥75 minutes vigorous or ≥150 minutes moderate activity per week	6.5	1.00	0.77–1.29	0.98	10.0	1.17	0.99–1.37	0.06
Transport-related physical activity									
	<75 minutes vigorous or <150 minutes moderate activity per week	6.7	1.00			11.1	1.00		
	≥75 minutes vigorous or ≥150 minutes moderate activity per week	6.5	0.89	0.73–1.09	0.26	8.6	1.00	0.86–1.16	0.95
**Co-morbidities**									
Angina pectoris									
	No	5.8	1.00			8.8	1.00		
	Yes	18.1	2.60	1.54–4.40	<0.001	25.2	2.21	1.75–2.80	<0.001
Diabetes									
	No	6.4	1.00			9.5	1.00		
	Yes	13.7	1.42	0.86–2.34	0.17	18.5	1.33	1.03–1.73	0.03
Chronic lung disease									
	No	5.7	1.00			9.0	1.00		
	Yes	15.9	1.70	1.30–2.24	<0.001	18.5	1.74	1.35–2.24	<0.001
Asthma									
	No	6.1	1.00			9.5	1.00		
	Yes	16.5	1.24	0.86–1.80	0.25	16.7	1.29	0.97–1.72	0.08
Depression									
	No	6.0	1.00			9.2	1.00		
	Yes	15.3	3.00	2.13–4.23	<0.001	19.0	1.85	1.53–2.24	<0.001
Hypertension									
	No	5.4	1.00			7.5	1.00		
	Yes	9.0	1.27	1.03–1.57	0.03	14.3	1.23	1.05–1.44	0.01
Stroke									
	No	6.5	1.00			9.7	1.00		
	Yes	12.7	1.09	0.73–1.65	0.67	18.5	1.12	0.77–1.64	0.55

* Post-stratified person-level weights applied

## Discussion

The use of gas, coal/charcoal, wood and agriculture/crop/animal dung/shrubs/grass for cooking was associated with an increased odds of arthritis compared to electricity in this study. Gender (female), older ages (50–59, 60–69, ≥70 years), overweight (25.0≤BMI<30 kg/m^2^) and obese (BMI≥30.0 kg/m^2^), former and current alcohol consumption, and having angina pectoris, diabetes, chronic lung disease, depression or hypertension were also associated with a higher odds of arthritis in the pooled analyses. Underweight (BMI<18.5 kg/m^2^), higher education (college/university completed/post-graduate studies) were associated with a lower odds of arthritis.

### Arthritis, ambient air pollution and smoking

The prevalence of arthritis in this study varied widely by country. Musculoskeletal diseases are the second leading causes of disability globally and osteoarthritis is associated has been associated with the greatest increases over the last 20 years.[39] Another study examining chronic diseases among older adults (>65 years) in Latin America (Cuba, Dominican Republic, Peru, Venezuela, Mexico), China and India reported an overall prevalence of arthritis comparable to that observed in the current study (18.2%)[40]. The prevalence of self-reported arthritis by country in urban and rural areas, respectively, was 14.5% and 22.3% (Mexico); 14.2% and 1.9% (China); and 18.2% and 51.1% (India)[22]. The reported prevalence among older adults (>65 years of age) from another meta-analysis study in South Africa was higher than that reported in this study (2.5% for rheumatoid arthritis; 29.5% to 82.7% for osteoarthritis) [41]. Similarly, the estimate of self-reported joint pain across the life course of adults (≥18 years) in a study of Russia was 44% [42]. Limited data regarding the prevalence of arthritis in Ghana outside of the SAGE studies were found.

Earlier studies have reported associations between ambient air pollution and RA. In a US-based study, Hart et al.[15] observed a 31% increased risk of incident RA among women living within 50m of primary (interstate and non-interstate) and secondary roads, though a second study did not observe an association with specific ambient pollutants.[18] Similar findings from a large study in Vancouver, Canada also showed a dose-response relationship with distance from highways or major roads and incident RA; however, no associations with specific pollutants were found except for ground level ozone.[16] Other studies have similarly observed associations between ambient air pollution and RA, RA biomarkers or clinical indicators (e.g. swollen joints) but not consistently with specific pollutants.[13, 14, 17, 43, 44]

Investigations of associations between ambient air pollution and arthritis have been preceded by studies focused on tobacco smoke and occupational health. Prior work has generally shown cigarette smoking to be a risk factor for the development and severity of RA.[25, 45–52] Conversely, smoking has been observed to have a protective effect in terms of hip, hand and knee OA in some, but not all studies, though the mechanism is unclear with confounding a potential issue.[28, 53–64] In this study, use of tobacco (former, current) was not found to be associated with arthritis in pooled or gender-stratified analyses. The proportion of women who used tobacco products in the study was very low, characteristic of low-income countries,[65–67] although the proportion of men who used tobacco in this study was relatively higher as compared to HIC.[67] In this study we did not specifically assess smoking but rather tobacco use, which also included snuff and chewing tobacco.[68]

### Arthritis and household air pollution

Use of gas, coal/charcoal, wood and agriculture/crop/animal dung/shrubs/grass fuels for cooking were positively associated with higher odds of arthritis compared to electricity. Only kerosene/paraffin was not associated with arthritis, though the proportion of users was very low (overall prevalence of 0.8%). These findings are congruent with others In a study in Stockholm, sulfur dioxide, modelled as a marker of home heating sources, was associated with an increased odds of RA in anti-citrullinated protein antibody-negative RA phenotype individuals with less than a university education.[18] Dahlgren et al.[69] also observed an increased odds of rheumatic diseases and systemic lupus erythematosus with exposure to volatiles, reduced sulfur compounds, heavy metals and house dust in New Mexico. Occupational exposures (mineral dust, insecticides, and textiles) have also been associated with an increased risk of RA.[45, 49, 70, 71] Studies investigating air pollution and OA are currently lacking.

Potential biological mechanisms linking air pollution exposure and autoimmune diseases like RA may be similar to those of smoking, which include oxidative stress and immune suppression.[16, 29, 30] Air pollution-induced oxidative stress may lead to the production of pro-inflammatory cytokines, promoting the maturation of airway-resident dendritic cells that migrate to lymph nodes and activate lymphocytes, triggering autoimmune responses in susceptible individuals.[72] Particulate matter may also promote disease via oxygen-free radical-generating activity, DNA oxidative damage and mutagenicity.[43] Potential biological mechanisms that may link osteoarthritis and air pollution are currently unexplored.[62, 73] However, proposed pathways include ozone-related vitamin D deficiency, the action of non-dioxin-like polychlorinated biphenyls or systemic inflammation leading to OA progression.[74–76] Studies of smoking have found an association with cartilage loss, which may also explain our findings.[31, 32] Exposure to air pollution from cooking activities is not likely to be transient, with exposures occurring throughout different life stages (in utero, infancy, childhood, adulthood),[77, 78] particularly in women, which may have ramifications in terms of the development of arthritis.

Cook fuel type may be an indicator of household socioeconomic status in LMIC,[79] which may be another explanation for the association between air pollution and arthritis, though adjustment for both education level and income quintile in this study did not attenuate this relationship. College/university or higher education was found to be protective for arthritis in this study, compared to those with primary school or less. Lower socioeconomic status and education levels independently have been associated with an increased risk of RA and OA in prior studies.[45, 80–84]

Repetitive joint stress from the collection and carrying of fuel, or the use of wood and charcoal stoves, which are typically lower to the ground and can involve sitting, kneeling, bowing or squatting,[85] may also partly explain this relationship. Squatting, bending and kneeling during cooking is typical when using stoves that burn biomass fuels in many regions.[86–89] The association among those who report using gas as the main fuel for cooking may be due to mixed stove and/or fuel use. Cooking is an activity that is conducted almost daily.[90–92] Sports, occupational or other socio-behavioural activities may be risk factors for OA related to the stress they place on joints.[93–98] A systematic review and dose-response meta-analysis found a 26% increased odds of OA per 5,000 hours of kneeling or squatting from pooled case-control studies that examined occupation-related exposures in men and women.[99] The impact of occupational and household activities requires further investigation.

### Arthritis and sociodemographic characteristics

In this study, older age and female gender were strongly associated with arthritis, which is consistent with the findings of several other studies.[13, 21, 100] We also observed a higher odds of arthritis among women classified as overweight and obese and lower odds among those classified as underweight, compared to those of normal weight. Obesity is a known risk factor for OA.[21, 45, 101–103] Although a complex relationship exists between arthritis and obesity, increased force on articular cartilage and subsequent breakdown can result from higher body weight.[104] No significant associations between arthritis and participants’ country were observed, with the exception of Mexico. The lower odds observed in Mexico could be due to underreporting or regional variations (within Mexico) in the reporting of arthritis.[105]

### Arthritis and lifestyle characteristics

The former and current consumption of alcohol among men was also associated with a higher odds of arthritis, compared to those who never drank. A systematic review and meta-analysis found that low to moderate alcohol consumption was associated with a lower risk of RA in both men and women.[106] Magnusson et al.[61] also observed that alcohol consumption was associated with current joint inflammation among those with hand OA. The positive association may also be related to the occurrence of gout, the most common inflammatory arthritis among men. Choi et al. observed increasing risk of gout with increasing levels of alcohol consumption in a US-based study.[107]

### Arthritis and comorbidities

In our study, we also observed that arthritis was associated with a number of different comorbidities, including angina pectoris, diabetes, chronic lung disease, depression and hypertension. Greater numbers of comorbidities have been found to be associated with RA and OA in other studies. [21, 36, 108–111] Multimorbidity is also associated with older age and being female.[111, 112] There is also emerging evidence for the independent role of metabolic syndromes (e.g. diabetes, hypertension, obesity) in the development of osteoarthritis.[75, 113]

### Strengths and limitations

We had the opportunity to examine household air pollution and other factors in relation to arthritis. A strength of this study is its focus on LMIC, understudied populations, and use of large datasets for the analyses. However, there are several limitations that that need to be considered together with the interpretation of these findings. Importantly, we were unable to distinguish between the different types of arthritis in this study, as diagnoses relied on self-reports and questions rather than physician- and/or laboratory-confirmed diagnoses. Validation studies of some self-reported measures against gold standard laboratory tests were planned for later waves but not available in Wave I. Symptoms of other non-arthritic conditions such as fibromyalgia can present similarly to arthritis.[114] Variability in terms of the level of accuracy in the reporting of different chronic diseases among elderly populations has been previously observed.[115–118] The diagnosis of arthritis is often difficult without the aid of other clinical, radiological or biological measurements, which are often lacking in LMIC settings.[23, 119–122] However, the sensitivity and specificity of combinations of answers regarding arthritis were checked against a Diagnostic Item Probability Study (2003) carried out as part of the validation of symptom questions used in the diagnosis of chronic diseases the first wave of the World Health Surveys.[123] Here on the primary focus of our discussions have concerned OA and RA, two of the most common types of arthritis, as studies of other types are scarce. Globally, OA is also the most prevalent type of arthritis.[19]

A limitation in air pollution studies concerns the assessment of exposures. In this study, the use of the primary type of fuel used for cooking is a proxy for household air pollution exposure. The assumption is that solid biomass fuels produce levels of household air pollution greater than gas fuels, though mixed fuel and stove use by households is likely more typical. [124–126] Information about mixed fuel and stove use was not available in the data. Thus, the associations reported here may neglect other critical exposure aspects. Further, data limitations prevented us from refining these exposures based on the type of stove used, presence of a chimney or hood and the location of cooking activities, though these variables were available in the dataset. Of those that responded, 83.4% reported using an open stove or fire for cooking, 43.5% reported the presence of a chimney or hood and 58.7% reported cooking in a separate room of the house. Multiple imputation was not undertaken due to a combination of factors related to the proportion of missing data (~50%) and indeterminate missing data mechanisms and patterns.[127, 128]

We were also unable to consider potential effects from ambient air pollution, which may be a significant exposure source in some of these regions, or other forms of household air pollution, such as those stemming from boiling water (other than for cooking) as well as heating and lighting activities.[1, 129, 130] We included setting (urban vs. rural) in our models as a proxy indicator for ambient air pollution exposure and did not observe an association, though analyses with more refined measures are warranted. Additionally, we cannot rule out the plausible possibility of reverse causality, given the study’s cross-sectional design. Those with arthritis may be unable to work or reduce their working hours, resulting in reduced income, which can have ramifications in terms of the type of fuels used in households.[131]

Cumulative or past exposures may be an important consideration in the development of arthritis. Questions about type of fuel used in this study focused on those mainly used in the household. As such, we are unable to draw any definitive conclusions as to the temporal relationships between household air pollution exposure and the development of arthritis. Air pollution may also be associated with the progression of the disease or worsening of symptoms.

## Conclusions

This study is among the first to examine the association between household air pollution and arthritis within LMICs, where the burden of these exposures is typically highest. Findings suggest that exposure to household air pollution is associated with an increased odds of arthritis. Future work focused on prospectively measured levels of household and ambient air pollution in these settings, biomarkers of exposure and exposures in relation to the development and progression of different types of arthritis is recommended.
